# Challenges in Diagnosis and Management of Spontaneous Coronary Artery Dissection in a Young Patient

**DOI:** 10.21470/1678-9741-2018-0198

**Published:** 2019

**Authors:** Beatriz J. S. Branco, Cesar Sanchez, Cesar Mendoza, Michael Magarakis, Alejandro Eric Macias, Tomas A. Salerno

**Affiliations:** 1 University of Mogi das Cruzes School of Medicine, São Paulo, Brazil.; 2 Ross University School of Medicine Ringgold standard institution, North Brunswick, New Jersey, United States.; 3 Department of Medicine; Division of Cardiology, Jackson Memorial Hospital, University of Miami Miller School of Medicine - Miami, Florida, United States.; 4 Division of Cardiothoracic Surgery; Cardiac Surgery Section, University of Miami Miller School of Medicine/Jackson Memorial Hospital, Miami, Florida, United States.; 5 University of Medicine and Health Sciences - Surgery, New York, United States.

**Keywords:** Vascular Diseases - Diagnosis, Coronary Artery Bypass, Off Pump - Methods, Young Adults

## Abstract

Spontaneous coronary artery dissection (SCAD) is characterized by tear of the inner layer in the coronary artery, creating a false lumen between the inner and central layer. Its infrequent incidence often leads to delay in diagnosis posing challenges in management. There are currently no guidelines for the treatment of this condition. We describe an adult patient who presented with multiple episodes of ventricular fibrillation, in whom cardiac catheterization showed SCAD, treated by off-pump coronary artery bypass.

**Table:** 

Abbreviations, acronyms & symbols	
ACS	= Acute coronary syndrome
CABG	= Coronary artery bypass graft
CPR	= Cardiopulmonary resuscitation
LAD	= Left anterior descending
LIMA	= Left internal mammary artery
PCI	= Percutaneous coronary intervention
SCAD	= Spontaneous coronary artery dissection

## INTRODUCTION

Spontaneous coronary artery dissection (SCAD) is an infrequent cause of acute coronary syndrome, occurring most frequently in young women. The treatment is mostly based on the clinical presentation^[1]^. We, herein, present a patient who sustained two episodes of ventricular fibrillation, who was successfully resuscitated and treated with off-pump coronary artery bypass surgery.

## CASE REPORT

A 36 year-old woman with no significant past medical history complained of crushing chest pain followed by collapse while deboarding a cruise ship. She received immediate cardiopulmonary resuscitation (CPR) at the scene and was found to be in ventricular fibrillation. Heart rhythm was recovered after defibrillation. However, she sustained a second episode of ventricular fibrillation requiring CPR for 15 minutes. She was successfully defibrillated with resumption of sinus rhythm and normal blood pressure. 

**Fig. 1:**
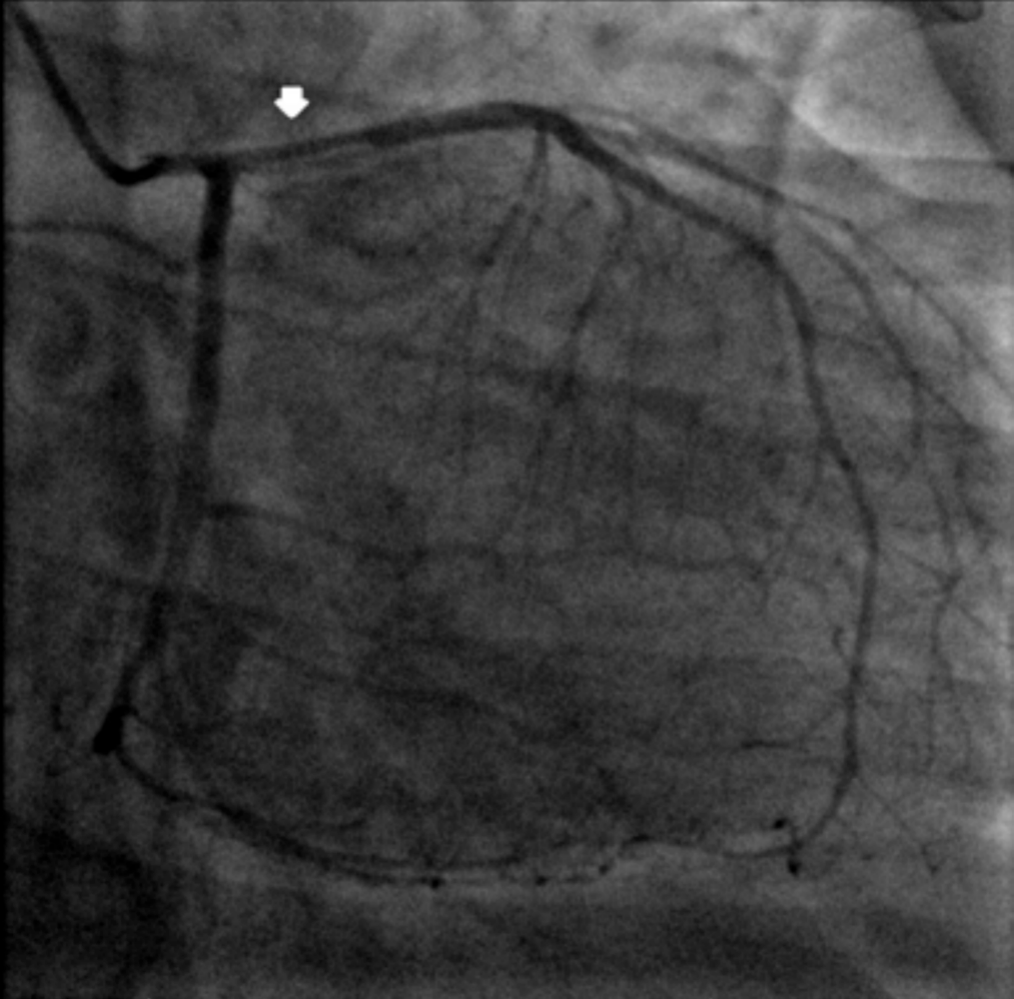
Spontaneous coronary artery dissection of the left anterior descending coronary artery (arrow).

The patient was air-lifted to our hospital, where her electrocardiogram showed sinus rhythm with no ST elevation. Transthoracic echocardiogram showed ejection fraction of 35 to 40%, with severe apical-septal hypokinesis and moderate to severe anterior wall hypokinesis. She underwent emergent cardiac catheterization, which showed SCAD involving the left anterior descending (LAD) coronary artery (Figure 1). 

**Fig. 2:**
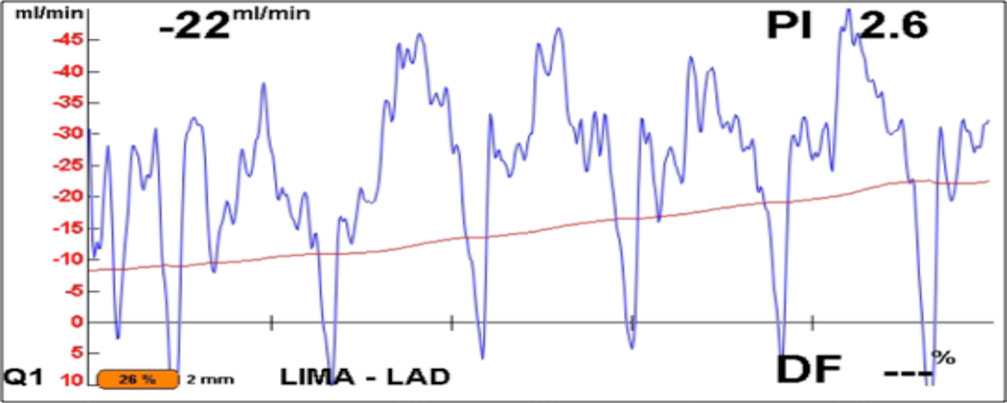
Flowmetry of the LIMA-LAD graft at the end of the surgical procedure.

There were considerable multidisciplinary discussions with cardiologists, surgeons and interventionists as to the best approach to treat this patient. Several factors were considered: her young age, complexity of the LAD dissection, the risk of percutaneous coronary intervention (PCI) if acute occlusion occurred during insertion of the stent, and stent stenosis in this young patient. Due to her previous two episodes of ventricular fibrillation, and the nature of the dissection of the LAD, it was decided to proceed with emergent off-pump coronary artery bypass graft. The left internal mammary artery (LIMA) was anastomosed to the LAD, and high diastolic flow, via flowmeter, was confirmed at the time of chest closure (Figure 2). 

A 12-lead electrocardiogram performed on post-op day one showed normal sinus rhythm. The patient made an uneventful recovery and was discharged on post-op day four wearing a cardioverter defibrillator LifeVest (ZOLL, Pittsburgh, PA), until evaluation by an electrophysiologist at her one month follow up. At follow up, there were no indications for insertion of an automated implantable cardioverter defibrillator, as she had no arrhythmias and normal left ventricular function, and the LifeVest (ZOLL, Pittsburgh, PA) was discontinued.

## DISCUSSION

SCAD is a spontaneous tear of the inner layer in the coronary artery, creating a false lumen between the inner and central layer. This results in decreased coronary blood flow leading to acute coronary syndrome (ACS)^[1,2]^. The first reported case of SCAD was in 1931 during an autopsy in a 42-year-old woman. The literature remains limited with information on SCAD^[3,4]^. Of all causes of ACS known in the general population, SCAD is responsible for only 0.1-0.4%. However, in the young female population (<50 years), SCAD is responsible for up to 25% of ACS^[1,3]^, mostly unrelated to pregnant women with low cardiovascular risks^[5]^.

SCAD is divided into atherosclerotic and non-atherosclerotic types: Atherosclerotic SCAD is characterized by rupture of an atherosclerotic plaque; non-atherosclerotic SCAD is related to other pathophysiologies, such as connective tissue disorders, systemic inflammation, coronary vasospasm, severe hypertension, physical stress, use of oral hormonal contraceptive, cocaine abuse, peripartum, and up to 72% due to fibromuscular dysplasia^[2-4]^. 

Though the diagnosis of SCAD remains challenging, it should be considered in any young female presenting with ACS symptoms, with or without ST segment elevation^[2]^. A recent study with 168 patients with SCAD revealed that only 26.1% of patients presented with ST-segment elevation, and 3.6% had ventricular fibrillation or ventricular tachycardia^[4]^. The LAD was found to be the most prevailing site of SCAD, as in our patient^[2,6,7]^.

A study in 2013 using Optical Coherence Tomography showed that up to 4% of all ACS were caused by SCAD^[3]^. Coronary angiography remains the standard diagnostic method due to its wide availability, except when dissection is caused by trauma during cardiac catheterization^[8]^. 

Saw 2014 defined three types of angiographic classifications of SCAD. Type 1 must include the pathognomonic appearance of having an arterial wall stain from contrast dye with multiple radiolucent lumen; Type 2 is often misdiagnosed and is characterized by diffuse stenosis of varying severity. This lesion can vary from subtle stenosis to complete occlusion; and Type 3 is the most difficult type to diagnose, as it closely resembles atherosclerosis. SCAD may be differentiated from atherosclerosis by lack of atherosclerotic changes in other coronary arteries, long lesions of 11-20 mm, angiographic hazy-appearing stenosis, and linear stenosis. Intracoronary imaging is imperative to identify such specific characteristics of a SCAD lesion^[9]^.

Currently, there are no guidelines for the treatment of SCAD. Once the diagnosis is made, treatment via percutaneous intervention or revascularization surgery depends on coronary anatomy and hemodynamic stability^[4,5,7]^. The majority of studies showed that SCAD lesions spontaneously resolved when patients were treated conservatively^[2,5]^. However, some patients required aggressive intervention during long-term follow up^[1]^. A study of 440 patients who presented with SCAD revealed 21.2% of patients who were treated conservatively required percutaneous coronary intervention (PCI) or surgical treatment, compared to 2.5% of those who were initially treated aggressively^[10]^. The authors demonstrated statistically significant larger symptom-free periods and lower mortality in the CABG and PCI groups compared to the medical management group.

Off pump coronary artery bypass surgery has been previously reported in the treatment of SCAD, especially when there is evidence of stenosis in other coronaries. In this way, it can serve to reduce the risk of aortic dissection due to manipulation of the aorta, or to avoid fluctuations in arterial pressure^[11-14]^. Furthermore, vascular pathology in these patients often involves the ascending aorta; a common cannulation site for the effectuation of cardiopulmonary bypass, which may become the point of future dissection^[14]^. The incidence of recurrence of coronary events in patients with SCAD is 17-20%, and the prognosis for these patients remains obscure^[8,11]^. 

## CONCLUSION

We report a young patient with two episodes of ventricular fibrillation successfully treated with CPR and defibrillation, who subsequently was found to have SCAD involving the LAD. In this particular patient, off pump coronary artery bypass was used to treat the patient with successful LIMA-LAD bypass. Currently, the literature reflects conservative medical management for patients with SCAD who are hemodynamically stable with no history of ischemic symptoms; early invasive intervention with PCI or CABG is recommended for patients with hemodynamic instability with or without a history of ischemic symptoms, or in patients who have recurrent angina. The best treatment approach must be individualized for each patient presenting with SCAD, as there are no current guidelines for its treatment.

**Table:** 

Authors' roles & responsibilities	
BJSB	Acquisition and analysis of data; conception and study design; manuscript redaction and revisal of content; agreement to be accountable for all aspects of the work in ensuring that questions related to the accuracy or integrity of any part of the work are appropriately investigated and resolved; final approval of the version to be published
CS	Acquisition and analysis of data; conception and study design; manuscript redaction and revisal of content; agreement to be accountable for all aspects of the work in ensuring that questions related to the accuracy or integrity of any part of the work are appropriately investigated and resolved; final approval of the version to be published
CM	Manuscript redaction and revisal of critical intellectual content; final approval of the version to be published; agreement to be accountable for all aspects of the work in ensuring that questions related to the accuracy or integrity of any part of the work are appropriately investigated and resolved; final approval of the version to be published
MM	Acquisition and analysis of data; conception and study design; manuscript redaction and revisal of content; agreement to be accountable for all aspects of the work in ensuring that questions related to the accuracy or integrity of any part of the work are appropriately investigated and resolved; final approval of the version to be published
AEM	Acquisition and analysis of data; conception and study design; manuscript redaction and revisal of content; agreement to be accountable for all aspects of the work in ensuring that questions related to the accuracy or integrity of any part of the work are appropriately investigated and resolved; final approval of the version to be published
TAS	Acquisition and analysis of data; conception and study design; manuscript redaction and revisal of content; agreement to be accountable for all aspects of the work in ensuring that questions related to the accuracy or integrity of any part of the work are appropriately investigated and resolved; final approval of the version to be published
